# The Effects of Neurotropic B Vitamins, Vitamin D3, and Alpha-Lipoic Acid in In Vitro Models of Neurodegeneration

**DOI:** 10.3390/nu18152555

**Published:** 2026-08-05

**Authors:** Christian Viel, Ying Shi, Ryan P. Trueman, Arnaud J. Ruiz, Melissa L. D. Rayner

**Affiliations:** 1 German Innovation Center, P&G Health Germany GmbH, Sulzbacher Straße 40, 65824 Schwalbach am Taunus, Germany; viel.c@pg.com; 2 Department of Pharmacology, UCL School of Pharmacy, University College London, 29-39 Brunswick Square, London WC1N 1AX, UK; ying_shi.22@ucl.ac.uk (Y.S.); r.tru3man@gmail.com (R.P.T.); a.ruiz@ucl.ac.uk (A.J.R.); 3 UCL Centre for Nerve Engineering, UCL School of Pharmacy, 29-39 Brunswick Square, London WC1N 1AX, UK

**Keywords:** peripheral neuropathy, neurodegeneration, nerve regeneration, homocysteine, vitamin B1, B6, B12, vitamin D3, alpha-lipoic acid

## Abstract

Background: Peripheral neuropathy (PN) is one of the most common disorders of the peripheral nervous system, affecting around 10% of the general adult population. PN results in pain, paraesthesia, and sensory loss, negatively impacting patients’ quality of life. The condition is characterised by damage to peripheral nerves and is often linked to risk factors, among which diabetes is the most common cause. The incidence rate of patients suffering from PN has significantly increased in the last three decades, yet there are currently no pharmacological treatments to reverse or prevent the development of PN. Therefore, neurotropic B vitamins, specifically vitamins B1, B6, and B12, which are essential for protecting nerves from damage, nerve regeneration, and maintaining nerve health, are promising treatment options. Other biofactors with therapeutic potential are vitamin D3, which has been described as a regulator of neuroplasticity and neuroinflammation with indications to promote neuronal regeneration following injury, as well as alpha-lipoic acid (ALA), a well-described treatment option in PN. Methods: The regenerative capacity of vitamins B1, B6, B12, and D3 and ALA was determined following neurite degeneration in vitro induced by either hydrogen peroxide (H_2_O_2_) or homocysteine. Results: In line with previous studies, the results show that a combination of vitamins B1, B6, and B12 has a significantly better effect on neurite extension following homocysteine-induced degeneration. In H_2_O_2_-insulted neural cells, the combination of all three neurotropic B vitamins was also shown to be superior to that of individual B vitamin treatment, demonstrated by longer neurite length after insult. Vitamin D3 also demonstrated a neuro-regenerative effect on neurites in healthy and insulted neural cells, whereas ALA only appears to provide a protective effect, preventing ongoing damage and supporting neurite outgrowth in healthy cells. No synergistic effects were seen with any treatment combinations. Conclusions: We have established an in vitro model of homocysteine-induced degeneration, and using this model alongside H_2_O_2_-induced degeneration, we provide evidence of the neurite regenerative capacity of vitamins B1, B6, B12, and D3.

## 1. Introduction

Peripheral neuropathy (PN), a prevalent neurological disorder, arises from numerous aetiologies, including autoimmune disorders, exposure to toxic agents, such as chemotherapeutic drugs, deficiencies in essential nutrients, such as vitamin B12, viral infections, inflammatory conditions, and metabolic disorders, including diabetes mellitus, affecting around 10% of the general adult population [1,2,3,4]. The mechanisms underlying PN are multifaceted, involving axonal degeneration [5], demyelination [6], ion channel dysregulation [7,8], central sensitisation [9], and neuroinflammation [10]. Currently, there are no pharmacological treatments to reverse or prevent the development of PN [11], emphasising the need for a comprehensive approach to treatment that addresses these underlying mechanisms.

Notably, diabetes mellitus is one of the most common causes of PN. Approximately 50% of adults with type 2 diabetes are affected by PN over the span of the disease [12]. Diabetic peripheral neuropathy (DPN) primarily manifests as a result of prolonged uncontrolled hyperglycaemia, which leads to a spectrum of neuropathic changes in the peripheral nerves. The pathogenesis of DPN is complex, involving metabolic, vascular, and genetic factors that collectively contribute to nerve damage and dysfunction. Although it is not regarded as the sole driver of nerve damage, hyperglycaemia and the subsequent disturbance of carbohydrate metabolism, inducing oxidative stress, further enhanced by advanced glycation end products (AGEs), play a major role [13,14,15,16,17]. The clinical presentation of DPN is highly variable, encompassing a range of symptoms, starting with sensory disturbances, such as burning, prickling, and numbness [1], and escalating into severe motor and autonomic dysfunctions, including severe pain, motor dysfunction, foot ulcers, and, in severe instances, amputation of the lower extremities. DPN can also cause motor weakness and loss of coordination, resulting in an increased risk of falls and disability [18]. Therefore, early diagnosis and effective treatment options that go beyond glucose control and symptomatic therapy in conjunction with lifestyle modification are essential for reducing both the intensity and incidence of DPN [11,19,20].

Currently, various in vivo models, including streptozotocin-induced diabetic rats [21,22], mice [23,24], and Chinese hamsters [25], are employed to investigate the pathogenesis of DPN. Despite their utility, these models have certain limitations, including masking details of transient molecular events and interactions in signalling pathways. Hence, in vitro models play a pivotal role in screening potential treatments, offering insights into therapeutic efficacy [26,27,28,29]. Monolayer cell cultures, one in vitro approach for modelling neurodegeneration, are advantageous compared to more complex systems, as they provide a simple and controlled environment, enabling the isolation and investigation of specific research questions [30].

Our previous investigations have elucidated methodologies for inducing neural degeneration in monolayer NG108-15 cells, encompassing exposure to suramin, the chemotherapeutic agent paclitaxel, or hydrogen peroxide (H_2_O_2_) [31]. The H_2_O_2_-induced degeneration model represents oxidative stress, which is the main driver of PN regardless of the underlying condition. Within this model, a combination of vitamins B1, B6, and B12 was found to significantly increase neurite growth following H_2_O_2_-induced degeneration [31]. As a result, we wanted to further utilise this model to screen other vitamins and substances for potential neuro-regenerative or neuroprotective effects, including vitamin D3 and alpha-lipoic acid (ALA).

Vitamin D3 has demonstrated positive effects in neuropathy therapy through a variety of mechanisms [32,33,34]. The active metabolic form of vitamin D3, 1,25-dihydroxy vitamin D3 (1,25(OH)2D3 or calcitriol), is a known regulator of genes involved in neuroplasticity, neuroprotection, neurotrophism, and neuroinflammation [35]. Vitamin D3 has been shown to support nerve regeneration and repair, which is crucial in managing neuropathy symptoms [36]. By maintaining calcium balance, vitamin D3 also improves neuronal function and supports nervous system health [37]. Although vitamin D3 has shown benefit in models of DPN, the exact mechanism of nerve regeneration is unclear.

ALA is an antioxidant naturally produced in the body; however, at higher doses, ALA is available in medicinal products to treat multiple conditions, including but not limited to obesity, DPN, neuropathic pain due to diabetes, and high cholesterol. ALA has been shown to be a powerful lipophilic free radical scavenger in vitro and in vivo, which is beneficial to treat oxidative stress [38]. In an animal model of DPN, ALA has corrected deficits in nerve conduction velocity and endoneural blood flow [39], and has been shown to protect peripheral nerves from ischemia [38].

The effect of ALA has also been explored extensively in human studies. A meta-analysis (1258 patients) exploring the effects of 600 mg of ALA intravenously administered over three weeks for diabetic polyneuropathy showed that ALA improved neuropathic symptoms and deficits [40]. Oral administration of ALA has also been shown to ameliorate neuropathic symptoms. A once-daily dose of 600 mg for five weeks improved pain, the Neuropathy Symptoms and Change (NSC) score, and the Patient Global Assessment of Efficacy (PGA) [41]. Despite the growing evidence of the benefit of ALA for neuropathy, further studies are necessary to determine its mechanistic properties.

Homocysteine is a key amino acid in the metabolism of methionine, linked to neurodegenerative diseases, such as dementia and PN [42,43,44]. Homocysteine levels increase with age primarily due to declining renal function, vitamin deficiencies (specifically B1, B6, B12, and folate), and a general slowing of metabolic processes. As a contributing factor to neurodegenerative pathologies, homocysteine is associated with a variety of deleterious mechanisms, including protein modifications, neurotransmission derangement, excitotoxicity, impaired metabolism, oxidative and endoplasmic reticulum stress, ischemic damage, neuroinflammation, and epigenetic changes [42,45]. A homocysteine-induced degeneration model is valuable to represent the pathophysiology of nerve damage in PN.

Within this study, we investigated both homocysteine- and H_2_O_2_-induced degeneration in mono- and co-culture in vitro models of NG108-15 neural cells and SCL4.1/F7 Schwann cells to screen for the regenerative capacity of vitamins D3, B1, B6, and B12 and ALA.

## 2. Materials and Methods

All materials were obtained from Sigma-Aldrich, Gillingham, UK, unless otherwise stated.

### 2.1. Cell Culture

NG108-15 cells, a hybrid neural cell line derived from mouse neuroblastoma and rat glioma, were cultured in Dulbecco’s Modified Eagle Medium (DMEM): F12 medium, supplemented with 10% *v*/*v* foetal bovine serum (FBS), 100 U/mL of penicillin, and 100 µg/mL of streptomycin in standard cell culture flasks. The cultures were maintained at sub-confluency at 37 °C with 5% CO_2_ and passaged when required. During neurite outgrowth assays, FBS was omitted from the medium.

SCL4.1/F7 cells (immortalised rat Schwann cell line) were cultured in high-glucose DMEM supplemented with 10% *v*/*v* FBS, 100 U/mL of penicillin, and 100 µg/mL of streptomycin, in standard cell culture flasks. The cultures were maintained at sub-confluency at 37 °C with 5% CO_2_ and passaged as required.

### 2.2. Preparation of Medium With and Without B Vitamins and Drug Treatments

A DMEM:F12 medium lacking vitamin B1, vitamin B6, and vitamin B12 was prepared (all other vitamins remained) as follows: for 500 mL of medium, 2250 mg D-glucose, 27.9 mg L-tyrosine disodium salt dihydrate, 13.1 mg L-serine, 9.38 mg glycine, 6.3 mg myo-inositol, 1.12 mg calcium-d-pantothenate, 4.49 mg choline chloride, 1.33 mg folic acid, and 0.025 mg iron(III) nitrate nonahydrate were added to 465 mL of Earle’s Balanced Salt Solution (EBSS) containing 20 mL of Minimum Essential Medium Eagle (MEM 10×), 10 mL of L-glutamine (200 mM; Thermo Scientific, Loughborough, UK), 100 U/mL of penicillin, and 100 µg/mL of streptomycin. This medium is referred to as ‘vitamin-B-free’ throughout the manuscript. Vitamins B1 (thiamine), B6 (pyridoxine), and B12 (cobalamin) were added individually or in combination, as indicated. Vitamin B combination refers to a dose of 40 μM vitamin B1, 20 μM vitamin B6, and 0.4 μM vitamin B12, as used previously in vitro on healthy mouse dorsal root ganglion (DRG) neurons [46] and NG108-15 cells [31,47,48]. When screening vitamin D3 (cholecalciferol) and ALA alone, they were added directly to the DMEM:F12 medium, and when used in combination with B vitamins, vitamin-B-free medium was utilised. Before deciphering the impact of vitamin D3 and ALA on damaged cells, we first needed to determine their effect on healthy cells. Initial dose–response analyses were conducted exposing NG108-15 neural cells to treatments of 0.001–10,000 nM of vitamin D3 or 0.1–100 μM of ALA for 72 h. The concentrations of vitamin D3 and ALA tested were chosen according to ranges reported within previous in vitro studies in the literature.

### 2.3. Seeding of Monolayer Cultures

NG108-15 and SCL4.1/F7 cells were seeded at a density of 5000 cells/well onto 96-well plates in FBS-free medium or high-glucose DMEM, respectively. Vitamin-B-free medium was used when vitamin B screening was conducted. Plates were loaded onto an Incucyte S3 (Sartorius, Göttingen, Germany) and analysed every 4 h to monitor neurite growth or cell number using phase-contrast imaging.

### 2.4. Fabrication of 3D Co-Cultures

Anisotropic cellular gels were prepared using 1 mL of solution containing 80% *v*/*v* Type I rat tail collagen (2 mg/mL in 0.6% acetic acid; First link UK Ltd., Birmingham, UK), 10% *v*/*v* MEM, 5.8% *v*/*v* sodium hydroxide, and 4.2% Schwann cell suspension (3 × 10^6^ SCL4.1/F7 cells per 1 mL gel) and were integrated with tethering mesh at opposite ends of a rectangular mould. Cellular gels were immersed in 12 mL of medium and incubated at 37 °C with 5% CO_2_ overnight to enable cellular self-alignment. These gels were then stabilised for 60 s. Each stabilised, aligned cellular gel was cut into 4 equal segments to allow higher throughput of treatment screening. Each gel segment was transferred to a separate well in a 24-well plate, then 100,000 NG108-15 cells were seeded on top of each segment.

### 2.5. Degeneration Induction

To determine the optimal concentration of homocysteine required to induce degeneration, a dose response was conducted (50–300 μM). The monolayer cell cultures were subjected to degeneration with either 10 μM of H_2_O_2_ for 2 h or 150 μM of homocysteine for 24 h. Following the insult, medium was removed and replaced with medium supplemented with vitamins B1, B6, and B12, vitamin D3, or ALA, or a combination of these.

### 2.6. Immunocytochemistry

Following fixation with 4% paraformaldehyde (PFA), monolayer cells or gels were washed three times with phosphate-buffered saline (PBS) and permeabilised using 0.5% Triton-X-100 in PBS. Washes were repeated before blocking using horse serum (1:20 in PBS; Vector Laboratories, Kirtlington, UK). Gels were washed before the addition of primary antibody (anti-βIII-Tubulin, Invitrogen, Paisley, UK) diluted (1:400) in PBS and incubated overnight at 4 °C. Washes were repeated before adding the corresponding secondary antibody (Dylight anti-mouse IgG 488) diluted (1:400) in PBS for 45 min at room temperature. The secondary antibody was washed, and the samples were stored at 4 °C in PBS before viewing. DAPI (1:1000) was used as a nuclei stain.

### 2.7. Image Analysis and Quantification

Phase-contrast images were captured using the Incucyte S3 at 20× magnification and fluorescence microscopy (Zeiss Axiolab A1, Axiocam Cm1, Cambridge, UK) was used to capture images of neurites from predetermined fields of each gel using a 20× lens. All neurites in the determined fields were manually measured using the NeuronJ plugin on Fiji Image J version 1.54p [49].

### 2.8. Cell Number Quantification

Cell number was quantified using the automated cell counter tool on the Incucyte S3. All cells within four fields were quantified.

### 2.9. Lactate Dehydrogenase (LDH) Assay

The lactate dehydrogenase (LDH) assay (Abcam (ab65393), Cambridge, UK) was conducted using the manufacturer’s protocol to quantify LDH in the cell medium. A 10% Triton X-100 in PBS was used as a positive control. Absorbances were measured at 450 nm and 650 nm using a SpectraMax M2e plate reader (Molecular Devices Ltd., Berkshire, UK).

### 2.10. Statistical Analysis

Data are presented as mean ± standard error of the mean (SEM). Normality tests were conducted on all data to determine appropriate statistical tests, and an unpaired *t*-test or one-way ANOVA with a Dunnett’s or Tukey’s post hoc test was conducted, as the data followed a normal distribution. For all tests, * *p* < 0.05, ** *p* < 0.01, *** *p* < 0.001, and **** *p* < 0.0001 were considered to be significant. All figures were created with GraphPad Prism version 10.1.0.

## 3. Results

### 3.1. Non-Insulted Monolayer NG108-15 Cells Treated with B Vitamins

**Figure 1:**
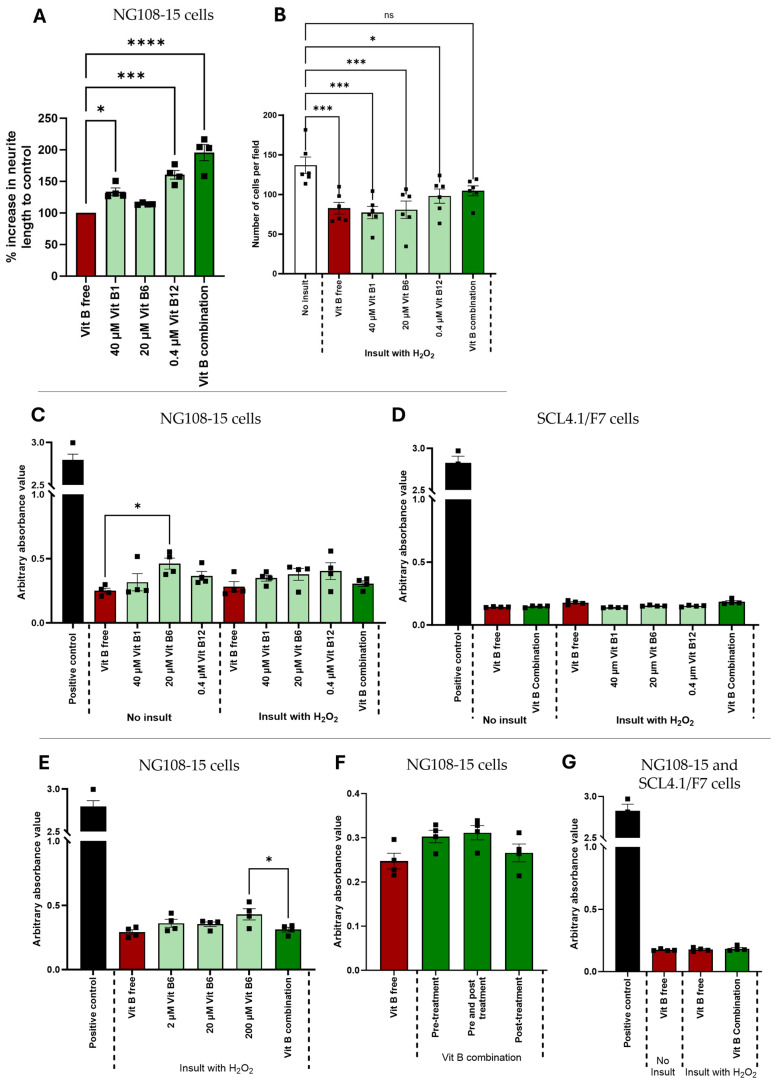
(**A**) Neurite length increased in comparison to the control when monolayer NG108-15 cells were treated with vitamins B1, B6, and B12 in isolation or combination. (**B**) The number of NG108-15 cells was significantly reduced when degenerated with hydrogen peroxide (H_2_O_2_) in comparison to the non-insulted cells, except in the group treated with vitamins B1, B6, and B12 in combination. In the LDH assays, the absorbance was lower in the experimental NG108-15 (**C**) and SCL4.1/F7 (**D**) cell groups than in the positive control, indicating reduced LDH release. Treatment with 20 μM vitamin B6 resulted in a significant increase in absorbance in comparison to the vitamin-B-free control in the non-insulted cells (**D**). A further dose response of vitamin B6 in the insulted cells showed that 200 μM vitamin B6 significantly increased the absorbance (**E**). No difference in absorbance was observed when NG108-15 cells were treated pre- or post-insult (**F**) or when co-cultured with SCL4.1/F7 cells (**G**). *n* = 4 (in triplicate; *n*= 6 in (**B**)). Mean ± SEM. One-way ANOVA followed by a Dunnett’s or Tukey’s post hoc test. ns = non-significant, * *p* < 0.05, *** *p* < 0.001, and **** *p* < 0.0001. Vit = vitamin.

As previously demonstrated [31,48], vitamins B1, B6, and B12 alone or in combination also increased neurite length in comparison to the vitamin-B-free control in the present study. There is a significant synergistic effect when all three B vitamins are given in combination rather than separately. The combination treatment resulted in a 95.63 ± 25.50% (mean ± SD) increase in neurite length, which was statistically significant in comparison to the 33.96 ± 11.29%, 14.27 ± 1.65%, and 60.59 ± 13.68% increases seen with vitamins B1, B6, and B12, respectively (Figure 1A). Besides the combination treatment, the vitamin B12 only treatment was the only other group that significantly increased neurite length.

### 3.2. Insulted Monolayer NG108-15 Treated with B Vitamins

To gain further insight into the regenerative effects of B vitamins, NG108-15 cells were insulted with H_2_O_2_ to induce degeneration, and cell number and viability were analysed. The number of NG108-15 cells was significantly lower when treated with individual B vitamins (−43.60% for B1, −40.96% for B6, and −28.41% for B12) following degeneration with H_2_O_2_ in comparison to the non-insulted cells, but in the NG108-15 cell group treated with vitamins B1, B6, and B12 in combination, the cell count reduction was not significant (−23.57%; Figure 1B). Compared to the combination treatment with three B vitamins, the reduction in cell counts for individual B vitamins were −26.21% for B1, −22.75% for B6, and −6.33% for B12.

### 3.3. Insulted Monolayer of SCL4.1/F7 Schwann Cells Treated with B Vitamins

The effect of B vitamins on SCL4.1/F7 Schwann cells was also tested. The cell counts of SCL4.1/F7 were significantly reduced after being exposed to H_2_O_2_ compared with non-insulted cells (Supplementary Figure S1). Again, the cell number was highest in the insulted cells treated with the combination of B1, B6, and B12 compared to the cells treated only with individual B vitamins.

### 3.4. LDH Assay for Cytotoxicity

In the LDH assays, the absorbance obtained from the 10% Triton-X-100-positive control, a non-ionic detergent permeabilising the cells, was significantly higher (*p* < 0.0001) than that of the experimental groups, indicating a greater LDH release from the cells, which is assumed to correspond to greater cell death. The absorbances for the experimental NG108-15 and SCL4.1/F7 cell groups that were insulted with H_2_O_2_ were not significantly different to the non-insulted groups (Figure 1C,D). A further dose response of vitamin B6 in the insulted cells showed that at a concentration of 200 μM, vitamin B6 significantly increased the absorbance value (Figure 1E). No difference in absorbance was seen with pre- or post-treatment of the B vitamin combination for insulted NG108-15 cells (Figure 1F). In the co-culture of NG108-15 and SCL4.1/F7 cells, no significant differences in absorbance between undamaged and treated and untreated insulted cells were observed (Figure 1G).

Following the significant effects on neurite growth observed with the B vitamins, we wanted to explore the effects of other vitamins and biofactors as possible therapies for PN. Vitamin D3 and ALA have both demonstrated beneficial effects in previous literature and were explored in this study [40,41,50].

### 3.5. Effect of Vitamin D3 and Alpha-Lipoic Acid (ALA) on Healthy NG108-15 Cells

**Figure 2:**
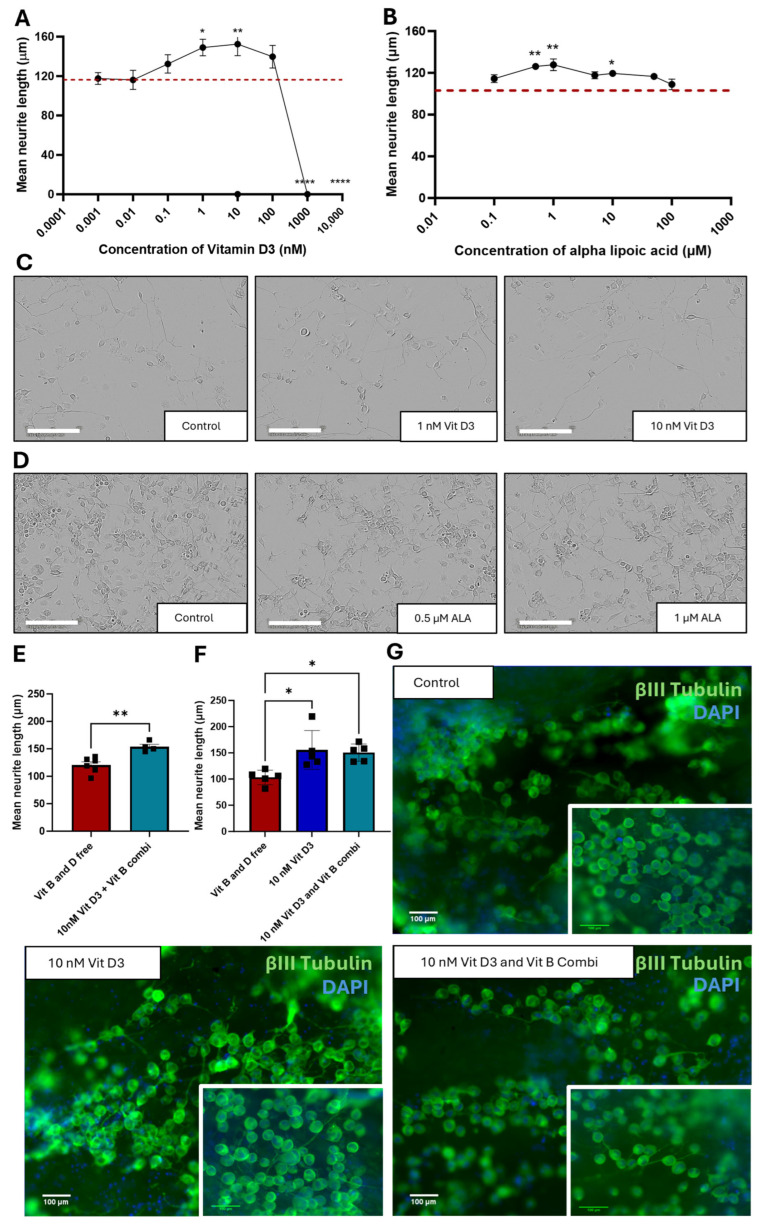
(**A**) A dose response in healthy monolayer NG108-15 cells demonstrated that vitamin D3 significantly increased neurite growth at 1 and 10 nM and ablated neurite growth at 1000 and 10,000 nM. (**B**) A dose response in healthy monolayer NG108-15 cells demonstrated that alpha-lipoic acid (ALA) had a significant effect on neurite growth at 0.5, 1 μM, and 10 μM. No treatment controls shown by red dotted lines. *n* = 8, mean ± SEM. One-way ANOVA followed by a Dunnett’s post hoc test. Phase-contrast micrographs showing neurite growth following 3 days of vitamin D3 (**C**) and ALA (**D**) treatment in monolayer NG108-15 cells obtained at ×20 magnification on the Incucyte S3. Scale bar = 200 μm. A significant increase in neurite length was seen when a combination treatment of 10 nM vitamin D3 and vitamin B combination (40 μM B1, 20 μM B6, and 0.4 μM B12) was given in both the monolayer cultures (**E**) and co-cultures (**F**), but no synergistic effect was observed. (**E**) NG108-15 cells; *n* = 4 (in quadruplicate), mean ± SEM. Unpaired *t*-test. (**F**) NG108-15 and SCL4.1/F7 cells; *n* = 5, mean ± SEM. One-way ANOVA followed by a Dunnett’s post hoc test. (**G**) Fluorescent micrographs of 3D co-cultures immunostained with βIII tubulin (green) and DAPI nuclei stain (blue) at 10× magnification (inset 20× magnification). Scale bar = 100 μm. * *p* < 0.05, ** *p* < 0.01, and **** *p* < 0.0001. Vit = vitamin.

Dose responses of vitamin D3 and ALA were conducted on healthy NG108-15 cells to find suitable and effective concentrations for further investigations. Vitamin D3 had a significant effect on neurite outgrowth at 1 nM and 10 nM with increases of 27.82% and 37.60%, respectively (Figure 2A,C). A 10-fold higher concentration did not lead to an increase in neurite length; on the contrary, neurite length decreased compared to the concentration of 1 nM vitamin D3, with cell death seen at 1000 and 10,000 nM (Figure 2A). For further investigations with vitamin D3, a concentration of 10 nM was used, as it had the greatest effect on neurite outgrowth.

ALA had a significant effect on neurite outgrowth at 0.5 μM, 1 μM, and 10 μM with increases of 23.37%, 23.36%, and 15.81%, respectively (Figure 2B,D). At higher concentrations, however, ALA had no beneficial effect on neurite length (Figure 2B).

### 3.6. Effect of Vitamin D3 and B Vitamins on Healthy NG108-15 and SCL4.1/F7 Cells

Given the positive effects on neurite length observed with vitamin D3 in isolation, we wanted to explore whether combining it with B vitamins produced any synergistic effects. A significant increase in neurite length was evident when combining 10 nM of vitamin D3 with 40 μM of B1, 20 μM of B6, and 0.4 μM of B12, with a mean length of 153.72 ± 4.51 μm; however, there was no synergistic effect, as a treatment with 10 nM of vitamin D3 alone elicited a mean neurite length of 152.52 ± 11.77 μm (Figure 2A,E).

In the collagen co-culture system of NG108-15 and SCL4.1/F7 cells (Figure 2F,G), treatment with 10 nM of vitamin D3 increased neurite length by 50.58%, greater than that measured in the monolayer cultures (37.60%). As discovered in the monolayer cultures, the combination of 10 nM of vitamin D3 with 40 μM of B1, 20 μM of B6, and 0.4 μM of B12 increased neurite length (45.68%) with a mean length of 150.42 ± 16.46 μm in comparison to the non-treated group (103.26 ± 13.77 μm). However, no synergistic effect was observed, as treatment with vitamin D3 alone resulted in a mean neurite length of 155.48 ± 37.14 μm (Figure 2E,F).

### 3.7. Homocysteine-Induced Degeneration

**Figure 3:**
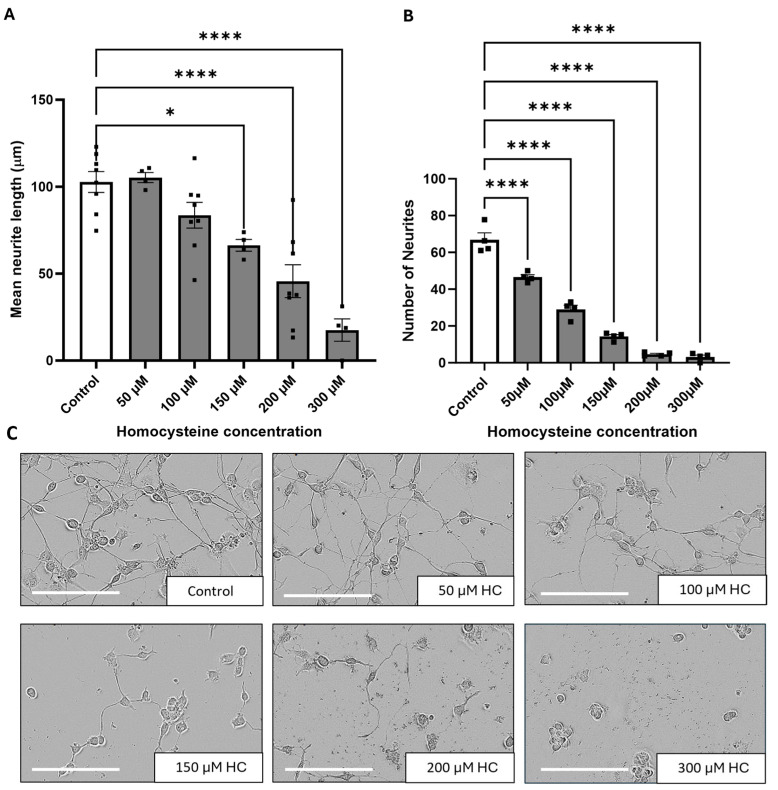
Neurite length (**A**) and number (**B**) were quantified at 72 h post-insult with increasing concentrations of homocysteine. Phase-contrast micrographs acquired using an Incucyte S3 demonstrating the degenerative effect of a dose range of 50–300 µM homocysteine (HC) doses on neurite growth in NG108-15 cells (**C**). *n* = 8 (in quadruplicate; control, 100 μM, and 200 μM) and *n* = 4 (in quadruplicate; 50 μM, 150 μM, and 300 μM). One-way ANOVA followed by a Dunnett’s post hoc test. * *p* < 0.05 and **** *p* < 0.0001. Scale bar = 200 μm.

NG108-15 cells were treated with a concentration range of 50–300 μM homocysteine, and the mean neurite length and neurite number were measured (Figure 3A–C). A concentration of 150 µM or higher induced a significant degenerative effect on the neurite length in a dose–response manner, with observed apoptosis at concentrations exceeding 200 µM (Figure 3A,C). The number of neurites, however, was more sensitive to homocysteine-induced degeneration in that a concentration of 50 µM significantly reduced the number of neurites (Figure 3B). A concentration of 150 μM was found to be optimal for inducing neurite degeneration without triggering significant cell death and was, therefore, used in future studies.

After a 24 h treatment with 150 μM of homocysteine or a 2 h treatment with 10 μM of H_2_O_2_, to induce degeneration as previously described [31], the cells were treated with vitamins B1, B6, B12, and D3 and ALA, or combinations of B vitamins and D3 and ALA, respectively, or left untreated for 24 h.

### 3.8. Regenerative Effect of Vitamins and ALA in Monolayer NG108-15 Cells with Homocysteine-Induced Degeneration

**Figure 4:**
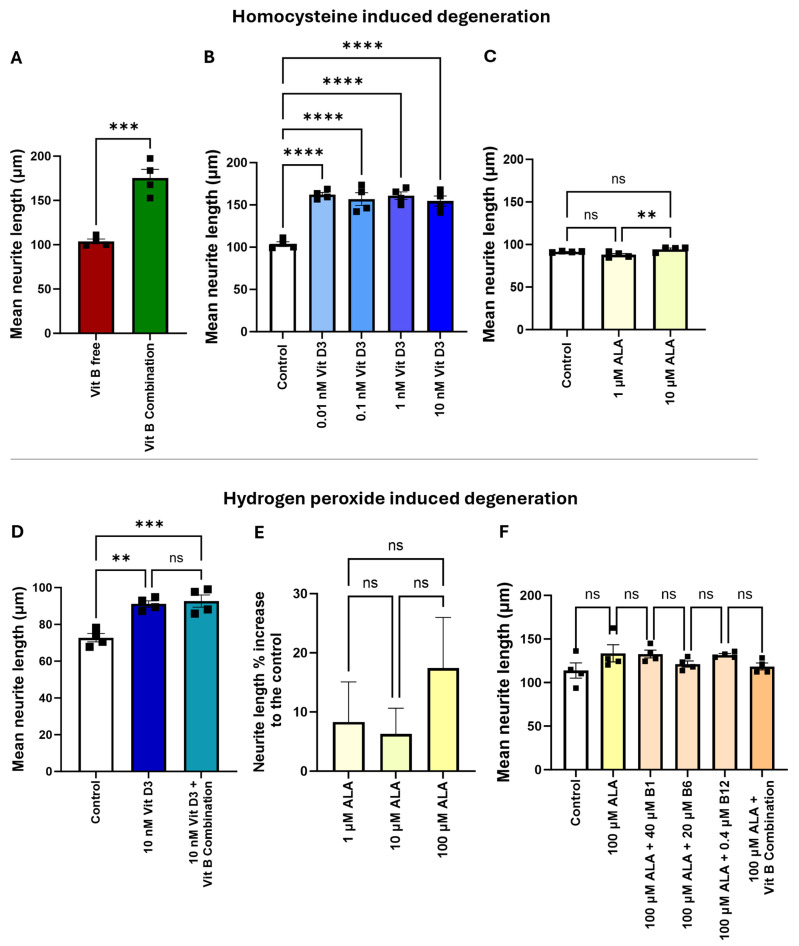
Mean neurite length significantly increased with vitamin B combination treatment following homocysteine-induced degeneration (**A**) or various concentrations of vitamin D3 (**B**); however, no benefit was seen with alpha-lipoic acid (ALA) (**C**). Similarly, mean neurite length significantly increased following hydrogen-peroxide-induced degeneration with vitamin D3; however, it produced no synergistic effect, as was seen when combined with the B vitamins (**D**). Also, treatment with ALA did not result in a significant increase in neurite length and even seemed to ablate the previous effect seen with B vitamins (**E**,**F**). Control = induced degeneration without treatment; *n* = 4 (in quadruplicate), mean ± SEM. Unpaired *t*-test or one-way ANOVA followed by a Dunnett’s or Tukey’s post hoc test. ns = non-significant. ** *p* < 0.01, *** *p* < 0.001, and **** *p* < 0.0001. Vit = vitamin.

Following homocysteine-induced degeneration, the combination of the three B vitamins (40 μM B1, 20 μM B6, and 0.4 μM B12) significantly (*p* = 0.0004) increased the mean neurite length in comparison to a vitamin-B-free control (69.11%; Figure 4A). A concentration range of 0.01–10 nM vitamin D3 was screened, with a neurite length increase seen at all concentrations (Figure 4B). On the other hand, when the cells were treated with 1 or 10 μM of ALA, selected due to the increase in neurite length observed at these concentrations in the healthy cells, no difference in the neurite length was seen in comparison to the non-treated homocysteine-damaged control (Figure 4C).

### 3.9. Regenerative Effect of Vitamins and ALA in Monolayer NG108-15 Cells with Hydrogen-Peroxide-Induced Degeneration

Similar beneficial effects were observed in the H_2_O_2_-induced degeneration. Vitamin D3 resulted in a 25.21% increase in neurite length following H_2_O_2_ insult. When vitamin D3 was combined with the three B vitamins, a significant increase in neurite length in comparison to the control remained; however, there was no difference to vitamin D3 treatment alone, with an increase of 27.38% (Figure 4D). Similar to the previous results, 1 or 10 μM of ALA did not have any significant regenerative effect on mean neurite length (Figure 4E). The effect of ALA on neurite outgrowth was not linear to the increase in concentration in that 10 µM of ALA did not lead to a greater mean neurite length than 1 µM of ALA. At a higher concentration of 100 μM of ALA, an increase of 17.40% was observed in neurite length; however, this was not significant (Figure 4E). Furthermore, when ALA was combined with all three B vitamins or individual B vitamins (i.e., B1, B6, or B12), no combination demonstrated a significant effect on neurite length compared to the untreated control or ALA only treatment (Figure 4F).

## 4. Discussion

Mitochondrial impairment, driven by chronic hyperglycaemia and overproduction of reactive oxygen species (ROS), including H_2_O_2_, is a defining feature of diabetic neuropathy, the most common of type of PN [51,52]. But also in PN of different aetiologies, inflammation, ROS, and H_2_O_2_ are main drivers of the pathophysiological mechanisms, i.e., chemotherapy-induced and alcohol-induced PN [53,54]. Chronic conditions like obesity and metabolic syndrome are also associated with PN and can be regarded as independent risk factors for the development of the disease [55]. H_2_O_2_-induced neuronal degeneration occurs through the activation of apoptotic pathways, particularly via caspase-3, a key executioner of apoptosis [51]. This contributes to the loss of sensory and motor neurons, which results in the characteristic symptoms of neuropathy, such as pain, paraesthesia, numbness, and loss of function [56,57]. Therefore, using H_2_O_2_ to create neurodegenerative in vitro models to study peripheral neuropathy is of relevance and efficacious [58].

### 4.1. Neurite Outgrowth and Cell Count

Our previous research has shown that treating the NG108-15 cell line with 5–10 µM of H_2_O_2_ for 2–4 h effectively induces neuronal degeneration [31,47]. Using this model, we showed that neurotropic B vitamins in combination have a superior effect over individual B vitamins on neurite regeneration following H_2_O_2_ insult [31,47]. The superiority of the B vitamin combination with regard to neurite outgrowth of non-insulted cells was reported in a previous study [48] and corroborated in this study. The combination of vitamins B1, B6, and B12 effectively supported the restoration of neurites (neurite length increased by 26%) compared to the untreated control (Figure 1A). Further to this, we observed that the combination of vitamins B1, B6, and B12 retained the number of cells following H_2_O_2_ so that the cell counts were not significantly different from non-insulted cells (Figure 1B). This indicates a cell-protective effect specific for the combination of vitamins B1, B6, and B12. Treatments with each B vitamin individually did not retain cell numbers as effectively as the combination of all three B vitamins. The effect of the H_2_O_2_ insult was also tested on Schwann cells alone in order to distinguish between the effect of the insult on either neuronal or Schwann cells in co-culture (Supplementary Figure S1). Schwann cells were dramatically affected by the H_2_O_2_ insult, indicating their sensitivity to oxidative stress. The combination of all three neurotropic B vitamins retained the most Schwann cells, although the effect was not significant when compared to each individual B vitamin treatment.

### 4.2. Lactate Dehydrogenase Assays for Cytotoxicity

LDH assays were performed to assess the cytotoxicity of B vitamins in neuronal as well as Schwann cells. No significant negative results were seen for any of the treatments after H_2_O_2_ insult (Figure 1C,D). We also observed no significant effects of pre- or post-treatment or both pre- and post-treatment with the B vitamin combination on insulted neural cells (Figure 1F). In co-culture, the combination of neurotropic B vitamins also had no significant effects detectable by the LDH assay (Figure 1G).

With a 10-fold higher concentration of vitamin B6 (200 µM) than we had used and tested previously [31,47,48], a significant effect on the absorbance value compared to the neurotropic B vitamin combination was detected for damaged cells, indicating cell loss at high B6 concentrations. In relation to the positive control, this damage was still not substantial and relatively mild (Figure 1E). The reported cytotoxic effect of vitamin B6 on human neural cells [59] was also only confirmed at higher concentrations. The LDH assay clearly demonstrated that neither the B vitamins, including 20 µM of vitamin B6, nor the combination of all three B vitamins had cytotoxic effects on either of the neural cells. These test results are also relevant in that the safety of vitamin B6 intake is a recurring topic of discussion among experts [60,61,62]. Among the water-soluble B vitamins, vitamin B6 plays a special role, as long-term overdose can lead to neurological symptoms that closely resemble PN. An expert consensus on the therapeutic use of vitamin B6 was recently published [63], addressing the most important aspects of vitamin B6 use for patients.

### 4.3. Effect of Vitamin D3 and B Vitamins on Healthy NG108-15 and SCL4.1/F7 Cells

To find the optimal vitamin D3 concentration for neurite outgrowth, we subjected the neural cells to different vitamin D3 concentrations for 72 h and documented the corresponding neurite length, which turned out to be 10 nM (Figure 2A). With this vitamin D3 concentration we measured a mean neurite length of 152.52 ± 11.77 μm, demonstrating that vitamin D3 has significant effects on neurite outgrowth (Figure 2E). When vitamin D3 and the three neurotropic B vitamins were combined, no synergistic effect on neurite outgrowth was observed. The same general trends were observed in the co-culture; however, the percentual neurite length increase was 12.98% greater than in the monoculture when treated with 10 nM of vitamin D3 (37.60% vs. 50.58%, Figure 2F). This significant difference may be explainable with a Schwann-cell-mediated production of neurite growth factor (NGF). Through its genomic mode of action, vitamin D3 induces NGF expression in Schwann cells [64]. The increase in NGF supplied by the Schwann cells may stimulate neurite extension and would explain the enhanced neurite outgrowth observed only in the co-culture. As in the monoculture, vitamin D3 and neurotropic B vitamins combined did not elicit additional effects beyond those observed with vitamin D3 alone on neurite outgrowth (Figure 2F,G). We postulate two distinct biochemical and genomic modes of action (for vitamins B1, B6, and B12 and for vitamin D3) with very little direct interaction. In line with this hypothesis is the absence of a detectable synergistic effect when neurite length is used as the parameter and the combination of vitamins B1, B6, and B12 and vitamin D3 are given concomitantly. To our knowledge, this lack of synergy has not yet been described in any other neuronal in vitro model. It would be of interest whether this finding can be reproduced in other cell culture models. The current understanding of how vitamins B1, B6, and B12 exert their effects at the genomic level is incomplete. Although some preliminary observations suggest potential mechanisms, this area requires further studies [47]. One possible explanation for the absence of synergism is that vitamin D3 and neurotropic B vitamins primarily act through distinct molecular pathways. However, this could provide a perspective on a complementary therapy option in certain cases. Vitamin D3, through its active metabolite 1,25-dihydroxyvitamin D3 (calcitriol), functions largely as a neurosteroid hormone due to its ability to modulate gene expression, e.g., upregulation or downregulation of genes involved in neuronal survival, function, and repair. It can suppress pro-apoptotic genes and/or activate anti-apoptotic pathways, thereby enhancing neuronal survival under challenging conditions like hypoxia. Calcitriol can inhibit the production of pro-inflammatory cytokines, such as tumour necrosis factor alpha (TNF-α), interleukin-1 beta (IL-1β), and IL-6, and enhance the production of anti-inflammatory cytokines (e.g., IL-10). Inflammation is a significant driver of nerve damage in many neuropathies. By inhibiting neuroinflammation, vitamin D3 creates a less hostile environment, which is critical for nerve cell survival and for enabling subsequent repair processes. Reduced inflammation also minimises secondary damage to axons and myelin [50,65]. Vitamin D in general is an emerging therapy option for nerve-related conditions as well as deficiency in vitamin D is strongly correlated to development of peripheral neuropathy [66,67]. A single high-dosage administration of vitamin D3 effectively improved quality of life of patients with DPN [68]. Very high weekly doses of vitamin D3 for 12 weeks were effective in reducing symptoms and signs of DPN [69].

### 4.4. Homocysteine Insult for Neurodegeneration

**Figure 5:**
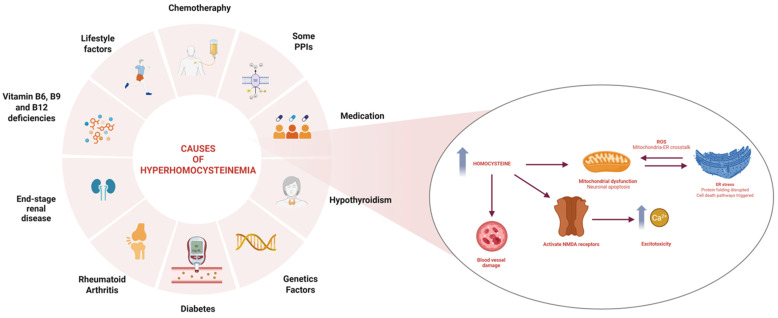
A schematic illustrating common causes of hyperhomocysteinemia and the mechanisms through which homocysteine causes nerve degeneration (Created in BioRender.com. Viel, C. (2026) https://app.biorender.com/citation/6a70bc8a50c56a0a487a2a9e).

Another aim of this study was to develop a new insult for neurodegeneration, as elevated homocysteine levels seem to be associated with PN [53] (Figure 5). Hyperhomocysteinemia is a common condition in patients with metabolic syndrome, the elderly population, obese patients, and other risk groups [70,71]. Increasing concentrations of homocysteine were screened for their effect on mean neurite length and number of neurites, with all concentrations causing a reduction in both (Figure 3A–C) and complete cell death seen at 300 μM. A concentration of 150 μM of homocysteine was used for subsequent studies because, although we observed a 46% reduction in mean neurite length and a 53% reduction in the number of neurites compared with a non-insulted control, there was no observed effect on cell viability.

These results align with earlier studies highlighting the neurotoxic potential of elevated homocysteine levels at concentrations of ≥100 μM [72,73]. Suggested mechanisms of homocysteine toxicity include intracellular calcium increase following excitotoxicity mediated by activation of the N-methyl-D-aspartate (NMDA) receptor, production of ROS, endoplasmic reticulum stress, and/or DNA damage [74]. Comprehensive pathway analysis has also suggested that homocysteine can modulate the expression of pro-inflammatory molecules, pro-apoptotic factors, signalling proteins, DNA, and macromolecule methylation enzymes, which could cause and/or enhance the progression of neurodegeneration [75].

The combination of the three neurotropic B vitamins was highly effective in recovering neurite length after homocysteine-induced degeneration (Figure 4A). Vitamin D3 was also very effective in recovering neurite length after homocysteine-induced degeneration. Interestingly, the effect was not concentration dependent, as 10 nM had no greater effect than a concentration three orders of magnitude lower (0.01 nM; Figure 4B). Exerting its effects by a genomic mode of action different from, for example, vitamins B1, B6, and B12 or ALA, the concentration of vitamin D3 is secondary. A very low concentration of vitamin D3 is sufficient to activate specific gene expressions mediated through the nuclear vitamin D receptor. Once this genomic mode of action is activated, higher concentrations of vitamin D3 cannot significantly contribute to an increase in specific gene expressions, as this appears to be an on–off phenomenon.

In contrast to that, ALA could not recover homocysteine-degenerated neural cells (Figure 4C). We also tested a 10-fold higher concentration, which yielded the same result. ALA, mainly an antioxidant, is not able to reverse the degeneration induced by homocysteine because it is not mainly caused by oxidative stress but by inflammatory and pro-apoptotic signalling. In this context, ALA primarily protects existing nerve structures from oxidative stress and mitigates ongoing damage [76], whereas neurotropic B vitamins actively support the process of nerve repair and regeneration. While both mechanisms of protection and regeneration are vital for nerve health, their modes of action are distinct, as exemplified by ALA versus B vitamins [77].

### 4.5. Hydrogen-Peroxide-Induced Degeneration

Similar to the results with homocysteine-degenerated neural cells, a concentration of 10 nM of vitamin D3 recovered neurite length effectively (Figure 4D). These findings are in agreement with numerous in vivo and clinical works attributing vitamin D3 an essential role in neuronal regeneration [32,34], although hard evidence in the area of PN is needed, namely, randomised clinical trials investigating the efficacy and dosage of vitamin D3 suitable for treatment of PN [78]. Combining neurotropic B vitamins with vitamin D3 did not demonstrate synergistic effects on neural cells because their primary mechanisms of action are largely independent, addressing different physiological pathways crucial for nerve health, and their individual optimal effects are achieved through distinct cellular processes that do not potentiate each other’s activity when co-administered. Neurotropic B vitamins directly support the biochemical machinery required for nerve cell structure, function, and repair. Their effects are primarily through enzyme catalysis and the synthesis of structural components (e.g., the myelin sheath). Vitamin D3 is a prohormone with a primary genomic mechanism of action, modulating neurotrophic factor synthesis (e.g., NGF), neuronal survival, and growth, suppressing pro-inflammatory cytokines, and indirectly reducing oxidative stress by upregulating antioxidant enzymes or pathways. While both types of vitamins are crucial for nerve health and regeneration, their molecular targets and direct biochemical interactions are largely distinct.

B vitamins facilitate specific enzymatic reactions and provide precursors, and vitamin D3 modulates gene expression. There is no direct biochemical step where, for instance, a B vitamin requires active vitamin D3 to perform its coenzyme function, or vice versa. In summary, this hypothesis suggests that B vitamins and vitamin D3 operate on distinct yet equally important biochemical pathways. Their mechanisms are complementary in that both are necessary for overall nerve health and regeneration, but not necessarily synergistic in a way that leads to a potentiated effect when combined.

In H_2_O_2_-degenerated neural cells, ALA was effective at different concentrations than we observed for healthy neural cells, where 100 µM negatively affected neurite outgrowth (Figure 2B). However, none of the effects were statistically significant. When subjected to oxidative stress, neural cells tolerate ALA in much higher concentrations. This suggests that there must be a biochemical need for ALA in order for higher doses to be tolerated. Additionally, such high concentrations were more effective at promoting neurite outgrowth (Figure 4E). ALA has been used in clinical practice for decades, and its mode of action is mainly based on antioxidative properties [79]. The herein presented findings are in line with this and emphasise the concentration-dependent antioxidative mode of action of ALA. A recently published animal study with a diabetic rat model found a protective effect of ALA, where streptozotocin-induced diabetic rats were orally administered with ALA one week after successful induction of the diabetic state. Energy supply in the axonal cell areas was enhanced by anterograde mitochondrial transport. The axonal areas are mostly the first neuronal parts to suffer oxidative damage. In this model, nerve conduction measures and behavioural tests also showed improvement [80].

Lastly, we tested combinations of B vitamins and ALA (Figure 4F). There was no significant difference in recovering neurite outgrowth after H_2_O_2_-induced degeneration between the individual B vitamins (B1, B6, and B12), the combination of all three B vitamins combined with ALA, and ALA alone. Compared to an untreated control, none of the effects were significant (Figure 4F). The absence of any effect is probably a direct molecular interaction of ALA and the B vitamins in the aqueous medium (Figure 4E,F). As a redox-active substance, it may oxidise B vitamins in these high concentrations (100 µM of ALA) used in the in vitro assays, leaving no intact B vitamins in the medium. Moreover, ALA and vitamin B1 can form a 1:1 complex in aqueous solution, inhibiting their individual physiological function [81].

### 4.6. Limitations and Outlook

Translating in vitro findings into clinical practice faces inherent limitations due to the lack of biological complexity in simplified in vitro models, and caution must be exercised in this regard, but they provide first evidence for the mode of action and whether there is to be expected a positive effect on neurons.

The use of immortalised cell lines is also a limitation, as they are genetically further away from functional neuronal tissue than primary cells lines and might not behave like them in regard to cell growth and resistance to toxic agents.

## 5. Conclusions

In conclusion, the models developed and utilised in our studies incorporate key features of neurodegeneration, highlighting their relevance for studying the mode of action for potential therapeutic candidates for peripheral neuropathy. Alongside the growing evidence of the superior effect of the combination of vitamins B1, B6, and B12 on extending neurite growth and regeneration in vitro, we have also demonstrated a neuro-regenerative effect with vitamin D3 in promoting neurite extension in both homocysteine- and hydrogen-peroxide-induced models of neurodegeneration. On the other hand, ALA did not demonstrate a neuro-regenerative effect but a potential neuroprotective effect, as it increased neurite length in healthy monolayer NG108-15 cells. Furthermore, we found that the combination of vitamin D3 or ALA with the neurotropic B vitamins demonstrated no synergistic effects in this model. Taken together, these findings demonstrate that combined neurotropic B vitamins and vitamin D3 promote neurite regeneration through distinct mechanisms, whereas ALA appears to exert neuroprotective rather than neuro-regenerative effects. Furthermore, the newly established homocysteine-induced degeneration model provides an additional in vitro platform for investigating therapeutic strategies targeting peripheral neuropathy.

## Data Availability

The original contributions presented in the study are included in the article/Supplementary Materials, further inquiries can be directed to the corresponding author.

## Supplementary Materials

The following supporting information can be downloaded at: https://www.mdpi.com/article/10.3390/nu18152555/s1. Figure S1: The number of SCL4.1/F7 cells was significantly reduced in all groups in comparison to the non-insulted cells. *n* = 6, mean ± SEM. One-way ANOVA followed by a Dunnett’s post hoc test. **** *p* < 0.0001.
