# Supplementary material for: The Effects of Neurotropic B Vitamins, Vitamin D3, and Alpha-Lipoic Acid in In Vitro Models of Neurodegeneration
Source: Nutrients. 2026 Aug 5;18(15):2555. doi: 10.3390/nu18152555 (PMC13468377; doi:10.3390/nu18152555)
Supplement: Supplementary file 1 [file nutrients-18-02555-s001.zip › nutrients-4429549-supplementary.pdf]

Supplementary Material

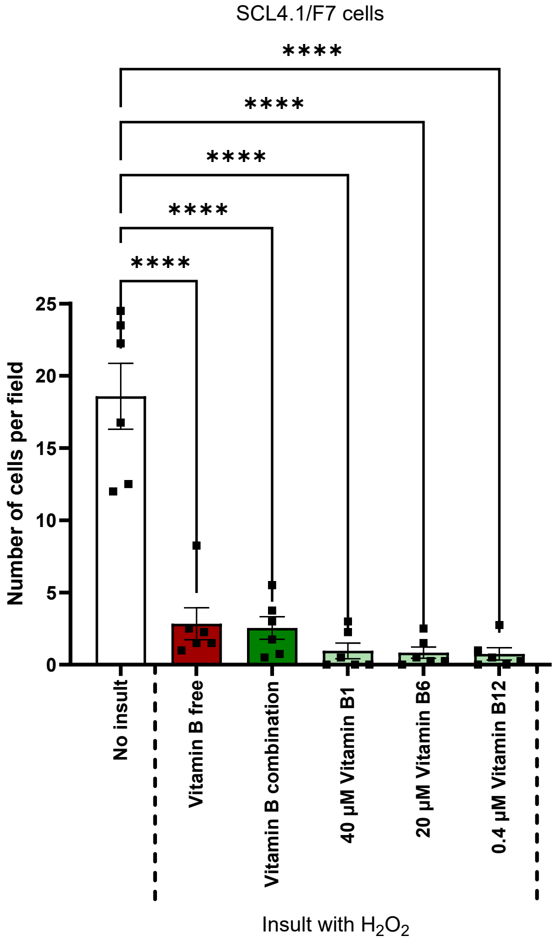

Supplementary Figure S1: The number of SCL4.1/F7 cells was significantly reduced in all groups in comparison to the non-insulted cells. N=6, mean ± SEM. One-way ANOVA followed by a Dunnett's post-hoc test. **** $p<0.0001$.
